# Inflammatory pseudotumor of the hip: a complication of arthroplasty to be recognized by the radiologist*

**DOI:** 10.1590/0100-3984.2013.0005

**Published:** 2015

**Authors:** Raquel de Melo Santos Vilas Boas, Ivana Andrade Madeira, Alexia Abuhid Lopes, Edson Barreto Paiva, André Soares Rodrigues

**Affiliations:** 1 Titular Members of Colégio Brasileiro de Radiologia e Diagnóstico por Imagem (CBR), MDs, Radiologists at Clínica Axial Medicina Diagnóstica, Belo Horizonte, MG, Brazil.; 2 Titular Member of Colégio Brasileiro de Radiologia e Diagnóstico por Imagem (CBR), MD, Radiologist, Specialist in Musculoskeletal System Imaging at Clínica Axial Medicina Diagnóstica, Belo Horizonte, MG, Brazil.; 3 Titular Members of Sociedade Brasileira de Quadril and Sociedade Brasileira de Ortopedia e Traumatologia, Hip Surgeons at Hospital das Clínicas – Universidade Federal de Minas Gerais (UFMG), Belo Horizonte, MG, Brazil.

**Keywords:** Magnetic resonance imaging, Inflammatory pseudotumor of the hip, Hip arthroplasty

## Abstract

Soft tissue complications following hip arthroplasty may occur either in cases of total hip arthroplasty or in hip resurfacing, a technique that has become popular in cases involving young patients. Both orthopedic and radiological literatures are now calling attention to these symptomatic periprosthetic soft tissue masses called inflammatory pseudotumors or aseptic lymphocytic vasculites-associated lesions. Pseudotumors are associated with pain, instability, neuropathy, and premature loosening of prosthetic components, frequently requiring early and difficult reoperation. Magnetic resonance imaging plays a relevant role in the evaluation of soft tissue changes in the painful hip after arthroplasty, ranging from early periprosthetic fluid collections to necrosis and more extensive tissue damage.

## Abstract (pt)

Complicações em partes moles pós-artroplastia do quadril são suscetíveis de ocorrer, seja quando da artroplastia total, seja quando se utiliza a técnica de recapeamento da cabeça femoral, opção que se tornou popular em casos de pacientes jovens. Tanto a literatura ortopédica quanto a radiológica têm chamado a atenção para massas “sintomáticas” que surgem em partes moles adjacentes a próteses, denominadas pseudotumores inflamatórios ou lesões associadas a vasculite linfocítica asséptica. Os pseudotumores estão associados a dor, instabilidade, neuropatia e afrouxamento prematuro dos componentes da prótese, geralmente levando a cirurgias de revisão precoces e difíceis. A ressonância magnética tem papel muito importante na avaliação das alterações em partes moles do quadril doloroso pós-artroplastia, que variam desde coleções fluidas periprotéticas precoces até necrose e dano tecidual mais extenso.

## INTRODUCTION

Soft tissue complications following hip arthroplasty may occur either in cases of total hip arthroplasty or in cases where the femoral head resurfacing technique is utilized.

Both the orthopedic and radiological literatures have highlighted the development of "symptomatic masses" in soft tissues adjacent to prosthesis, named pseudotumors, adverse reaction to metal debris, lesions associated with aseptic lymphocytic vasculitis, among others.

Inflammatory tumors affect principally patients with metal-on-metal surface prosthesis, but cases involving metal-on-polyethylene prosthesis have already been reported in the literature^(1-3)^. Historically, because of higher rates of complications, metal-on-metal prosthesis have been pushed aside after the arrival of polyethylene prosthesis^(4,5)^. Later, the creation of more durable prosthesis was required, mainly for younger and more active patients, so more modern metal-on-metal prosthesis were developed with the promise of lower indices of postoperative morbidity^(4-8)^.

The present study was aimed a reviewing of the literature, describing the main magnetic resonance imaging (MRI) findings in cases of such a complication associated with hip arthroplasty that must be recognized by the radiologist.

## DISCUSSION

The etiology of inflammatory pseudotumors still remains unknown, but it seems to be associated with a hypersensitivity reaction against metal and/or cytotoxic effect resulting from metal particles released by the prosthesis, predominantly affecting soft tissues, with development of periprosthetic cystic, solid or mixed masses, possibly leading to necrosis and a more extensive structural injury at long term^(4-6,9)^. Such a complication may develop months or even years after the surgical procedure^(3,4)^. The literature reports quite variable prevalence rates ranging from 14% to 36%^(6)^.

The symptoms are nonspecific and are not always present, including pain, joint instability, presence of a palpable mass, and association with adjacent neurovascular structures compromise^(7)^. There are reports on increased levels of chromium and cobalt in the blood, urine and joint fluid, but this finding does not define the diagnosis and is not observed in all the cases^(4,7,9-11)^.

Main risk factors reported in the literature include: female sex, young age (due to the greater prosthesis overload resulting from more intense activity); malpositioning of the prosthesis; and a reduced diameter of the femoral component^(4,5,9,10)^.

Main differential diagnoses to be considered include infection and neoplasm. C-reactive protein and erythrocyte sedimentation rate tests are useful to rule out the presence of infection^(9)^. At histology, the absence of neoplastic cells rules out neoplasia.

Histological findings include necrosis and dense perivascular lymphocytic infiltration into the surrounding viable tissue. The presence of metal particles is generally scarce, but such particles may be found within the macrophages^(4,6,8,9)^.

### Diagnostic evaluation

A series of recent studies published in Brazil have highlighted the relevance of imaging methods in the assessment of the musculoskeletal system^(12-24)^.

Plain radiography remains as the method of choice to assess the alignment and conditions of the prosthesis components, as well as the most commonly found complications such as osteolysis, fractures, heterotopic ossification, and prosthesis loosening^(4-7)^.

Ultrasonography is useful in the evaluation of fluid collections and masses, but is limited in cases of larger lesions or those located in deep planes^(4,5,7,8)^.

Computed tomography can detect large collections, but it is more frequently utilized complementarily with plain radiography to assess bone complications (osteolysis) and prosthesis positioning. The main limitations of this method include low contrast between soft tissues, which makes the identification of periprosthetic collections more difficult, and artifacts, that may be minimized with the use of multidetector apparatuses^(4,5,7)^.

**Figure 1:**
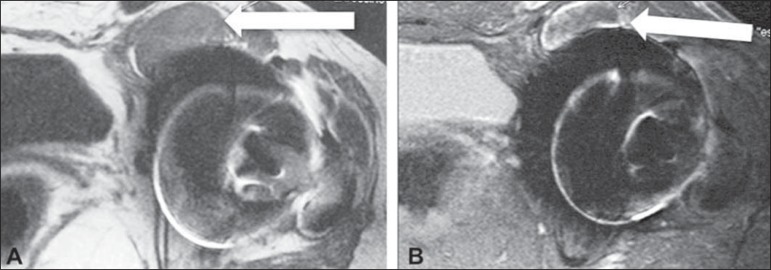
**A:** Axial, T1-weighted section demonstrating anterior collections adjacent to the joint (arrow), with hyperintense contents as compared with the bladder. **B:** Axial STIR section demonstrating heterogeneous collection with foci of hyposignal (arrow).

**Figure 2:**
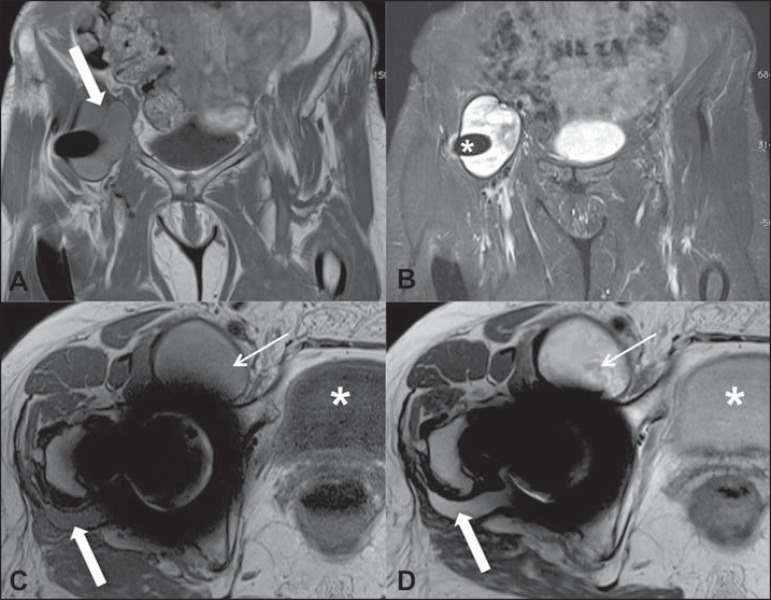
**A:** Coronal T1-weighted section showing the presence of a predominantly hyperintense collection (arrow) as compared with the bladder contents. **B:** Coronal STIR section demonstrating the presence of a predominantly hyperintense collection with a thin, smooth and hypointense capsule adjacent to thr prosthesis (asterisk). **C,D:** Axial, T1- and PD-weighted images showing an anterior collection (thin arrows). Compare the collection signal intensity with the bladder at the different sequences (asterisks). There is a small collection on the posterior aspect of the coxofemoral joint, with signal intensity similar to the anterior collection that should not be missed (gross arrows).

**Figure 3:**
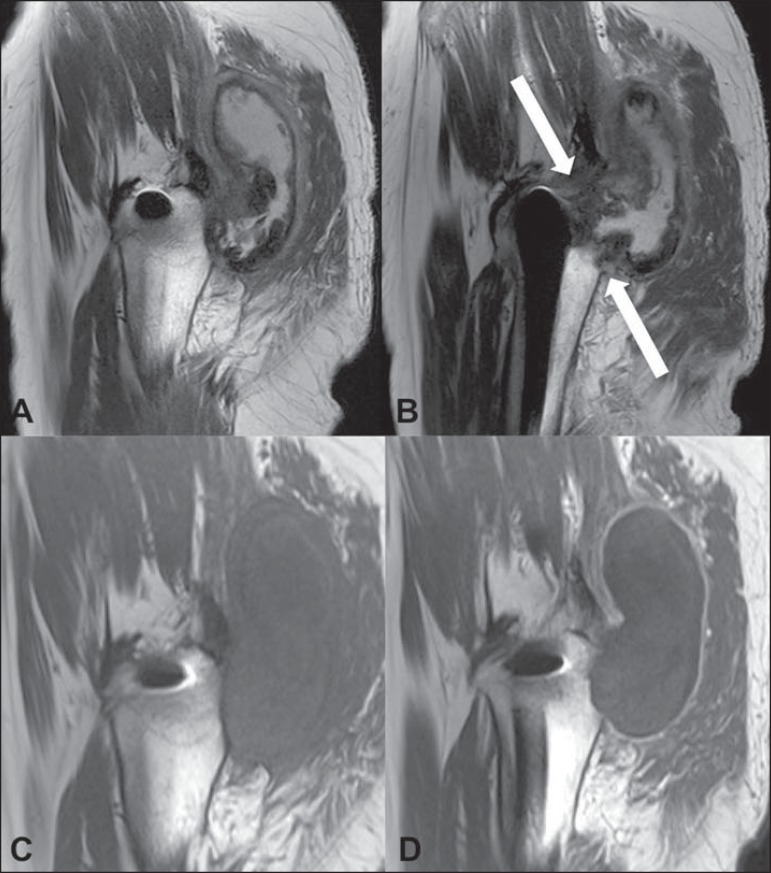
**A,B:** Sagittal sections showing posterior heterogeneous, predominalty hyperintense collection, with a thick and irregular capsule, extending toward the gluteal compartment. Observe the proximity with the joint (arrows). On the precontrast (**C**) and postcontrast (**D**) sagittal T1-weighted sections, observe posterior, collection isointense to the muscle with peripheral contrast uptake.

**Figure 4:**
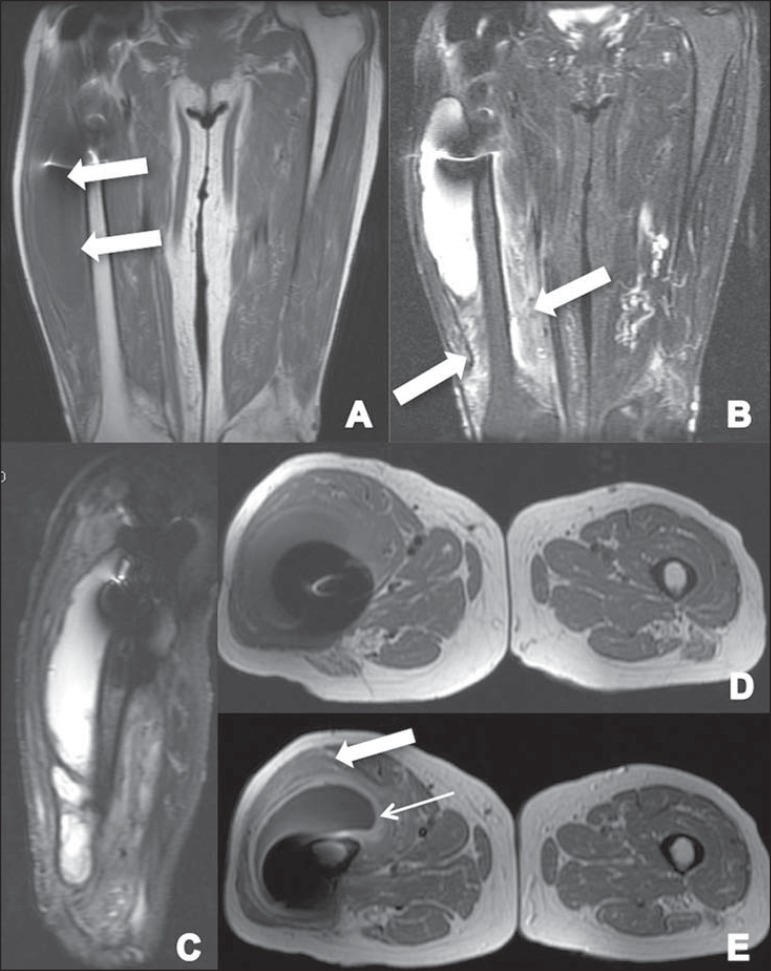
**A:** Coronal T1-weighted section showing the presence of a colleciton in the lateral aspect of the thigh, isointense to the muscle (arrows). **B:** Coronal STIR section showing hyperintense collection associated with edema on the thigh muscle (arrows). C: Sagittal STIR section showing hyperintense collection with a hypointense capsule in the anterolateral aspect of the thigh. **D,E:** Axial, T1-weighted sections acquired before and after intravenous contrast injection, showing the presence of a collection isointense to the muscle, with contrast uptake by the capsule (thin arrow) and by the muscle (gross arrow), the latter inferring the presence of muscle edema/myositis.

MRI has given a great contribution in the diagnosis of soft tissues complications, particularly in cases of pseudotumors, with the use of sequences which minimize magnetic susceptibility artifacts in 1.5 tesla apparatuses. At MRI, a pseudotumor appears like a collection, but sometimes it may appear like a solid mass in periprosthetic soft tissues^(4-7,9)^. The collection signal intensity is variable at T1-weighted sequences, most of times presenting a signal similar to the bladder contents suggestive of transudate^(9)^, or even a higher signal intensity than the one from the muscle, which is more specific, suggestive of complex exudate^(9)^. The signal intensity is also variable at T2- and PD-weighted sequences, generally hyperintense as compared with the muscle, and may be either homogeneous or heterogeneous (Figures 1 and 2). The hypointense content observed at those sequences may be related to the presence of necrosis or metal deposition^(4-6,9)^. Such sequences, together with STIR imaging, can better detect debris (Figure 2) and fluid-fluid level, as well as evaluate the capsule that is generally hypointense, either thin or thick, smooth or irregular (Figures 2 and 3). The solid mass is generally hypointense at T1- and T2-weighted sequences. Intravenous contrast injection is not required for the diagnosis, but in cases where it is used, peripheral uptake may be observed only in the lesion capsule (Figures 3 and 4)^(5,6,9)^.

The location is very characteristic, always adjacent to the prosthesis and to the joint (Figures 1, 2 and 3), generally related to the surgical route, so it is important to search for the area adjacent to the joint, which sometimes is difficult to identify (Figure 2)^(9)^.

A pseudotumor may extend toward adjacent compartments, namely, gluteal, adductor, quadricipital, peritrochanteric, anterior (proximal to the iliopsoas), posterior (ischiotibial) and subcutaneous compartments, through the deep fascia^(6,9)^.

The presence of associated myotendinous alterations should be reported to aid the orthopedist in a possible surgical reapproach, and includes tendinous avulsion, muscle edema resulting from early myositis (Figure 4), and muscle atrophy resulting from the surgery and lack of activity^(4,6,8,9)^.

Regional lymphadenopathy may be observed as a direct toxic effect of metal ions^(6)^. The involvement of adjacent neurovascular structures should also be carefully assessed since it migh result in neuropathy, stasis and/or thrombosis^(4,6,8)^.

## CONCLUSION

MRI is considered to be the main method for assessing soft tissues after hip arthroplasty, in spite of metal artifacts that can be minimized with the use of "large bandwidth" techniques increasingly better in terms of quality *versus* signal-noise rate.

The recognition of inflammatory pseudotumor by the radiologist becomes relevant considering the increasing number of surgical procedures and consequential postoperative complications.
